# Supplementary material for: Efficacy of prophylactic tranexamic acid among parturient at increased risk for postpartum hemorrhage undergoing cesarean delivery: A systematic review and meta-analyses of randomized controlled trials
Source: PLoS One. 2025 Oct 9;20(10):e0333177. doi: 10.1371/journal.pone.0333177 (PMC12510581; doi:10.1371/journal.pone.0333177)
Supplement: Attachment — Submitted filename: Reviewer response 2.docx [file pone.0333177.s003.docx]

Paris, July 2^nd^, 2025

Dear Jeremiah Hilkiah Wijaya, Academic Editor of *The PLOS ONE Journal*,

Firstly, we gratefully thank the reviewers for the carefully and rigorous reviewing of our work, which will undoubtedly improve the quality of our manuscript. As requested, please find attached the new version of the manuscript entitled: **“Efficacy of prophylactic tranexamic acid among parturient at increased risk for postpartum hemorrhage undergoing cesarean delivery: a systematic review and meta-analyses of randomized controlled trials**

**”.**

All criticisms raised by the reviewers have been adequately addressed and the respective changes have been highlighted in yellow-colored text in the revised manuscript.

Sincerely,

Dr Mohamed Aziz DAGHMOURI, on behalf of all co-authors.

**Reviewer #1**

**Reviewer:** Is the manuscript technically sound, and do the data support the conclusions? “Partly”

- **Response: We sincerely thank the reviewer for this important observation. We acknowledge that the data partially support the conclusions, as you rightly pointed out. In response, we have carefully revised the manuscript to ensure that the conclusions are now more closely aligned with the presented data. We believe these modifications improve the overall consistency and scientific soundness of the manuscript.**

**Reviewer:** This is a good article. A specific topic has been chosen, and few articles have been published on this topic. Based on the review of the results of the studies that have been conducted, could you give us some suggestions for improving future studies?

Good luck.

- **Response: We sincerely thank the reviewer for the positive feedback and thoughtful suggestion. As recommended, we have incorporated a dedicated section within the conclusion to outline directions for future research. These include the need for high-quality, large-scale randomized controlled trials, standardization of dosing regimens, identification of high-risk subpopulations, evaluation of long-term maternal and neonatal outcomes, and assessment of TXA's utility in resource-limited settings. We believe these additions provide a more comprehensive perspective and will help guide future studies in this field.**
- **“Conclusion: Prophylactic tranexamic acid appears to be a promising intervention for reducing intraoperative blood loss and the incidence of PPH in high-risk women undergoing cesarean deliveries. Our findings indicate that TXA is safe, well-tolerated, and cost-effective, with no significant increase in adverse effects. However, the current body of evidence remains limited in quality, necessitating further high-quality, large-scale randomized controlled trials to strengthen these conclusions. Future research should focus on standardizing dosing regimens, identifying specific subpopulations that may benefit most from TXA, and exploring long-term maternal and neonatal outcomes. Additionally, studies evaluating TXA’s impact in resource-limited settings, where access to blood transfusion and surgical interventions is constrained, would provide critical insights into its global applicability. Based on the available data, we advocate for the prophylactic administration of TXA in all women at high risk of bleeding during cesarean deliveries while emphasizing the need for continued research to refine guidelines and optimize patient outcomes.”**

**Reviewer #3**

**Reviewer:** Your manuscript is well-structured and methodologically sound, following PRISMA 2020 guidelines and PROSPERO registration. It effectively highlights the benefits of prophylactic tranexamic acid (TXA) in reducing intraoperative blood loss and postpartum hemorrhage (PPH) in high-risk cesarean deliveries

- **Response: We sincerely thank the reviewer for the encouraging feedback. We are pleased that the methodological rigor of our work, including adherence to PRISMA 2020 guidelines and PROSPERO registration, as well as the clarity of our findings on the benefits of prophylactic tranexamic acid in high-risk cesarean deliveries, have been positively noted. This recognition is greatly appreciated and reinforces our commitment to producing high-quality, evidence-based research.**
